# Editorial: Mineral solubilizing microorganisms (MSM) and their applications in nutrient availability, weathering and bioremediation

**DOI:** 10.3389/fmicb.2023.1101426

**Published:** 2023-01-24

**Authors:** Muhammad Zahid Mumtaz, Maqshoof Ahmad, Hassan Etesami, Adnan Mustafa

**Affiliations:** ^1^Institute of Molecular Biology and Biotechnology, The University of Lahore, Lahore, Pakistan; ^2^Department of Soil Science, The Islamia University of Bahawalpur, Bahawalpur, Pakistan; ^3^Department of Soil Science, College of Agriculture and Natural Resources, University of Tehran, Tehran, Iran; ^4^Faculty of Chemistry, Institute of Chemistry and Technology of Environmental Protection, University of Technology, Brno, Czechia; ^5^Department of Agrochemistry, Soil Science, Microbiology and Plant Nutrition, Faculty of AgriSciences, Mendel University in Brno, Brno, Czechia; ^6^Faculty of Science, Institute for Environmental Studies, Charles University in Prague, Prague, Czechia

**Keywords:** plant-microbe interactions, mineral-microbe interactions, minerals solubilization, nutrients availability, bioremediation

As the world's human population continues to grow, agricultural needs for future food supply will be one of the greatest challenges facing the agricultural community. In other words, agriculture is essential to achieve food security. Chemical fertilizers and pesticides have become a necessity in plant production to fulfill the rapid rise in population and, as a result, the increased nutritional needs. However, the unwanted and indiscriminate use of these fertilizers/pesticides causes many problems and has a negative impact on agricultural production in many countries today. In addition, soil pollution by chemical fertilizers, pesticides and heavy metals is a threat to the environment and food security due to the rapid growth of industry and agriculture and disruption of natural ecosystems by human pressures related to human population growth. Heavy metal pollution also poses many risks to the ecosystem and humans, affecting the safety of the food chain, food quality, and the ability to use land for agricultural production, which in turn affects food security. To meet this challenge, a lot of effort focusing on soil biological system and agro-ecosystem as a whole is needed to enable a better understanding of the complex processes and interactions among soil, plant and microorganisms governing the sustainability of agricultural land. Plant-associated microorganisms play a key role in solubilizing mineral substrates and contribute to the release of key nutrients from primary minerals and make essential plant elements available in soil, enhancing crop productivity (Etesami and Adl, 2020). In addition, these beneficial microbes are also involved in the degradation and/or detoxification of organic and inorganic compounds (bioremediation) present in the ecosystem (Etesami, 2018). Hence, introducing such a phytomicrobiome into the agricultural industry is an effective approach as a result of its long-term and environmentally favorable mechanisms to increase plant growth and preserve plant health and quality. In recent years, low-cost and environmentally friendly agricultural practices have received increasing attention.

This Research Topic, *Mineral-solubilizing microorganisms and their applications in nutrient availability, weathering and bioremediation*, presents one review paper and 12 original research papers, from 11 different countries (88 authors), and has papers that span the field of mineral-solubilizing microorganisms research, gives insight into ongoing topics, and provides a basis for further study on *Mineral-solubilizing microorganisms and their applications in nutrient availability, weathering and bioremediation*. Here, we summarized some of the highlights derived from the 13 articles published in this Research Topic.

Phosphorus (P) is one of the major plant nutrients, lack of which limits plant growth. Most agricultural soils contain large reserves of total P. Both P fixation and precipitation occur in soil because of the large reactivity of phosphate ions with numerous soil constituents. Developing microbial inoculants containing phosphate-solubilizing bacteria (PSB) represents an emerging biological solution to improve rhizosphere P availability (Etesami, 2020). In addition to PSB, the association of plants with mycorrhizal fungi defines a strategy to cope with P limitation (Plassard and Dell, 2010). The results of the study of Schreider et al. also provided evidence that an ectomycorrhiza has the potential to occupy fundamental niches of various P sources differing in their bioavailability, indicating that being a generalist in P nutrition can facilitate adaptation to various nutritional settings in soil.

Most publications describing isolation of PSB employed tricalcium phosphate. According to Bashan et al. (2013), tricalcium phosphate is relatively weak and unreliable as a universal selection factor for isolating and testing PSB for enhancing plant growth. A practical approach to screen true PSB is to use a combination of two or three metal-P compounds together. Janati et al. assessed several PSB strains' ability to enhance solubilization activity from rock phosphate, tricalcium phosphate, and their combination. The results of these researchers showed that the isolated bacteria had the ability to dissolve different sources of P both individually and in combination. It has been reported that the results obtained from the effect of PSB/plant growth promoting rhizobacteria (PGPR) on plant growth under axenic conditions could not be reproduced under field conditions (Smyth et al., 2011; Bashan et al., 2013). This might be occurred due to the low quality of the inoculums and/or the inability of the PSB/PGPR to compete with the indigenous population (Catroux et al., 2001) and to survive under stressful conditions (Etesami and Maheshwari, 2018). Janati et al. showed that the isolated PSB also were resistant to salinity, acidity, drought, and temperature stress, which can be used in stressful environments. Another reasons for the poor performance of agricultural bio-inocula in natural environments and in the rhizosphere of host plants could be the use of a single bacterial strain (van Veen et al., 1997). Khan M. Y. et al. showed that a multi-strain consortium of PGPR could be more effective to combat the salinity stress owing to the presence of a variety of plant growth-promoting traits (e.g., ACC-deaminase, phosphate solubilization, exopolysaccharides and siderophore). Compared to the well-known role of bacterial auxin hormone in increasing plant growth and resistance to stress, in the study of Zaheer et al., it was also found that bacterial cytokinin hormone also plays a significant role in plant growth.

It is known that rock/minerals weathering is the result of a combination of physical, chemical, and biological weathering, with organisms, particularly microorganisms, playing an important role in the early rock weathering process (Liu et al., 2020). Chen et al. provided new insights into the weathering process of carbonate rocks.

Zinc (Zn) is one of the most abundantly found heavy metals in the Earth's crust and is reported to be an essential trace metal required for the growth of living beings. However, its essentiality also runs parallel to its toxicity, which is induced through various anthropogenic sources, constant exposure to polluted sites, and other natural phenomena. Hussain et al. reviewed Zn and its properties, uses, bioavailability, toxicity, as well as the major mechanisms involved in its bioremediation from polluted soil and wastewaters. Dietary essential micronutrient (Zn, Fe, and Se) deficiency also affects a high percentage of the world population with significant health impacts. Microbial-assisted biofortification is a novel concept in the field of agricultural microbiology for nutrifying crop edibles. In the study of Upadhayay et al., the application of zinc-solubilizing bacteria caused an elevated Zn amount in the rice grain.

It has been a challenge to decontaminate industrial wastewater and soil from heavy metals and organic pollutants. Different strategies have been designed and implemented for their removal, including filtration, oxidation/reduction, reverse osmosis and electro-chemical treatment among many others, but are not the favored option due to their cost, inefficiency, and intense labor (Algieri et al., 2021). The use of bacteria possessing the ability to degrade organic pollutants, to immobilize heavy metals in soil and to promote plant growth, as single or in combination with plants, is an efficient and environmentally sustainable strategy to remediate organic pollutant and heavy metal-contaminated soils. Alotaibi et al. demonstrated that the combination of PGPR and the petroleum hydrocarbons (PHC) degradation potential of bacteria can result in an enhanced beneficial effect in phytoremediation management, which could lead to the development of innovative bacterial inoculants for plants to remediate PHC-contaminated soils. In the study of Khan M. et al., the potential of a copper (Cu)-resistant bacterium (*Bacillus altitudinis* MT422188) to remove Cu from polluted wastewater was investigated. Due to having some special features, *B. altitudinis* MT422188 was introduced as an efficient biosorbent for Cu that can be employed for Cu remediation. Xue et al. remedied copper-rich water bodies by using two-step biomineralization approach (i.e., securing the urease activity and modifying pH conditions).

In the study of Batool et al., the use of bacterial and fungal strains and zinc and iron-enriched rice husk biochar also resulted in maximum chromium [Cr(VI)] adsorption from wastewater applied to the soil by various mechanisms. In addition to bacteria and fungi, thermoacidophilic archaea also have a promising potential in bioleaching operations and metal recycling processes in regard to circular economies and waste management as investigated by Kölbl et al. To study microbial-assisted phytoremediation, the understanding the changes in the microbial community structure and the relationship between microbial community and soil environment is of utmost importance. Liu et al. studied microbial-assisted phytoremediation by exploring soil bacterial community. Their results showed that the diverse bacterial community is the result of adaptation to environmental changes.

## Author contributions

All authors listed have made a substantial, direct, and intellectual contribution to the work and approved it for publication.
